# Developing the Menstrual Myths Attitude Scale †

**DOI:** 10.3390/healthcare13182381

**Published:** 2025-09-22

**Authors:** Gülüzar Sade, Metin Yıldız

**Affiliations:** 1 Department of Midwifery, Faculty of Health Sciences, Tarsus University, Tarsus 33400, Turkey; guluzarsade@tarsus.edu.tr; 2 Department of Nursing, Faculty of Health Sciences, Sakarya University, Sakarya 54187, Turkey

**Keywords:** menstruation, menstrual cycle, menstrual myths, attitude, women

## Abstract

**Background:** Menstrual myths include practices that negatively impact women’s health. Knowing attitudes toward menstrual myths is essential for protecting and improving women’s health. However, there are limited measurement tools available to determine attitudes toward menstrual myths. **Methods:** This study is a methodological scale development study conducted between October 2021 and January 2023 to determine individuals’ attitudes towards menstrual myths. For this purpose, the scale items prepared by making use of the literature were shaped with expert opinions, pre-application was made, and data were collected from a total of 337 women through a questionnaire and validity analyses, including exploratory factor analysis (EFA), confirmatory factor analysis (CFA) and reliability analyses (Cronbach’s alpha), were also performed. **Results:** The final version of the scale included 20 items under 5 subscales (menstrual information, menstrual stigmatization, menstrual privacy, menstrual hygiene, menstruation education). Cronbach’s alpha coefficient was 0.79 for the whole scale. EFA revealed five factors explaining 52.074% of the total variance. CFA confirmed a good model fit: χ^2^ = 2876.480; df = 496, *p* < 0.05; X^2^/sd = 1.655; RMSEA = 0.044, AGFI = 0.910, IFI = 0.928, TLI = 0.913, GFI = 0.932, and CFI = 0.927. Cronbach’s alpha was 0.790 for the total scale. **Conclusions:** The Attitude Towards Menstrual Myths scale is a valid and reliable tool for determining women’s attitudes toward menstrual myths. It is recommended that further testing be conducted for use by different groups, men and gender-neutral individuals.

## 1. Introduction

Menstruation is a normal biological process experienced by millions of women and young girls worldwide every month. Menarche refers to the beginning of a woman’s reproductive years and is generally recognized as the transition to full adult female status in most societies [1]. Menstruation is the common source of various taboos and rituals in all traditional cultures. There are many myths and cultural misperceptions about menstruation that persist to this day [2].

In many societies, menstrual myths cause women to postpone personal care, feel ashamed, be left alone, have their physical activities inhibited, be stigmatized as dirty or sick, and negatively affect their spiritual lives. For example: during menstruation, Indian women do not wash their bodies, do not apply oil/cream, and do not apply make-up to their eyes. Until the end of menstruation, they sleep on the floor and do not sleep during the day, do not eat meat, cannot touch fire, cannot look at the planets and cannot clean their teeth [3]. In Ethiopia, girls perceive menstruation as dirty and shameful. They should not talk to men during menstruation, should not tell anyone that they are menstruating, and should live in secret [4]. In Zambia, girls are not asked to add salt to the food during menstruation because it is believed that the person who eats the food will have a chronic cough. Zambian women believe that menstrual bleeding will last longer when they do physical activity during menstruation, and that witches and satanists will use menstrual blood to bewitch and sterilize them [5]. In Turkish society, while birth, circumcision and wedding events are spoken aloud in public, talking about menstruation is perceived as shameful. In Turkish society, there is a generally negative attitude towards menstruation and persistent myths. Menstruating women generally express themselves as “I got dirty” or “I got sick” and must perform a purification ritual at the end of menstruation [6]. In many societies, myths about menstruation affect girls’ and women’s emotional states, mental functions, lifestyles and most importantly their health. Menstrual myths in different cultures cause girls to drop out of school, prevent them from transitioning to work, cause stigma within society, and lead to social isolation [7]. Menstrual myths restrict women’s daily activities and deprive them of hygienic health practices leading to negative consequences such as infection [8]. Ameade and Garti (2016) [9] reported in their research that girls are afraid and panic when they start their period. This suggests that girls lack knowledge about menstruation, affecting their general health and potentially leading to school absences. Chothe et al. (2014) [10] reported in their study that female students lack knowledge about the symptoms, irregularities, and myths surrounding menstruation. They reported that they do not consult a healthcare professional for menstrual irregularities and dysmenorrhea, but rather attempt to manage their problems themselves with traditional medications. This suggests that female students face difficulties accessing healthcare services. Research shows that during menstruation, adolescent girls change their eating habits, avoid cold foods, and limit their activities. This negatively impacts both their overall health and their growth and development [11,12]. Furthermore, one study found that menstruation is perceived as a shameful event, leading to social exclusion, isolation, and stigmatization [13]. Not showering during menstruation leads to poor hygiene and urogenital infections. Persistently, this situation leads to recurrent infections and sexual and reproductive health problems [7]. In their research, Akpenpuun and Azende (2014) [14] stated that girls’ knowledge level about menstruation is low, the myths they believe are many, and this is especially worrying in terms of reproductive health.

The difficulty of addressing socio-cultural myths, taboos and beliefs about menstruation prevents girls/women from receiving information on menstruation and reproductive health. In order to improve women’s/girls’ reproductive health, it is important to know their attitudes towards myths about menstruation. This is necessary in order to prepare strategies to combat myths. It is important to know menstrual myths and attitudes towards them in order for women and girls of reproductive age to experience menstruation healthily. Menstrual myths harm the physiological, psychological and spiritual health of menstruating people. To raise awareness of myths adopted by society and healthy practices during menstruation, individuals and families, healthcare professionals, educators, governments, politicians, and civil society organizations need to understand menstrual myths and their attitudes. The most reliable way to quantify people’s attitudes toward menstrual myths is through scales. During the literature review, it was determined that menstruation-related scales generally assess attitudes, beliefs, and the effects of menstruation [15,16,17,18]. However, a limited number of scales have been found to measure menstrual myths. The menstrual myths scale in the literature measures menstrual practices, beliefs, and personal hygiene practices [19]. However, this scale aims to measure myths related to menstrual information, menstrual stigmatization, menstrual privacy, menstrual hygiene, and menstruation education. Having multiple scales on the same topic would allow researchers to examine the topic more comprehensively. Furthermore, the diversity in the number of scale items, their adaptability to different cultures, and the suitability of the target population would allow for more flexible and appropriate measurements across different samples. Having different scales on the same topic would facilitate comparative analysis and provide more detailed information about their psychometric properties. This scale identifies menstrual myths and attitudes, and is believed to facilitate researchers in taking measures to address menstrual myths to improve women’s health and ensure women’s educational and employment continuity. In this context, the aim of this study was to develop a measurement tool to determine the attitudes of women of reproductive age toward menstrual myths.

## 2. Materials and Methods

### 2.1. Research Design and Sample

In this methodologically conducted study, data were collected through social media (WhatsApp, Facebook, and Instagram) and an online form from women of reproductive age (18–49 years old) who agreed to participate in the study between October 2021 and January 2023.

The study group of the research consists of 337 women of reproductive age reached by a convenience sampling method. The study included women aged 18–49 who spoke Turkish, used social media, and volunteered to participate in the study. Foreign national women, illiterate women, and menopausal women were excluded from the study. The scale was administered to 74 women for the pre-application and 337 women for Exploratory Factor Analysis (EFA) and Confirmatory Factor Analysis (CFA). In this context, descriptive statistics of the study group are given in Table 1.

According to Table 1, 57% of the women are married, 59.1% have higher education, 61.1% have income equal to expenses, and 70.3% live in the province.

### 2.2. Sample Size Justification

For a sufficient sample size for factor analysis in scale development studies, Bryman and Cramer [20] suggest that the number of items should be 5–10 times the number of items; Tabachnick and Fidell [21] suggest that the number of items is insignificant and recommends at least 300 individuals be included in the study. The study sample meets both criteria. The study’s sample size of 337 women demonstrates a sufficient level of validity and reliability.

### 2.3. Data Collection Tool

During the development of the scale, a literature review was first conducted. After the literature review [3,4,5,6,7,8,9,10,11,12,13,14], a 5-point Likert-type item pool consisting of a total of 32 items was created. Six people (two field experts, two scale development experts, and two Turkish language teachers) evaluated the prepared scale items, and three items were revised based on their feedback. The scale scores are Strongly Disagree = 1, Disagree = 2, Undecided = 3, Agree = 4, and Strongly Agree = 5. There are no reverse items in the scale. The highest score that the person to whom the scale is applied can get is 160 and the lowest score is 32. The increase in the score in the scale shows that the attitude towards menstrual myths increases.

**Figure 1:**
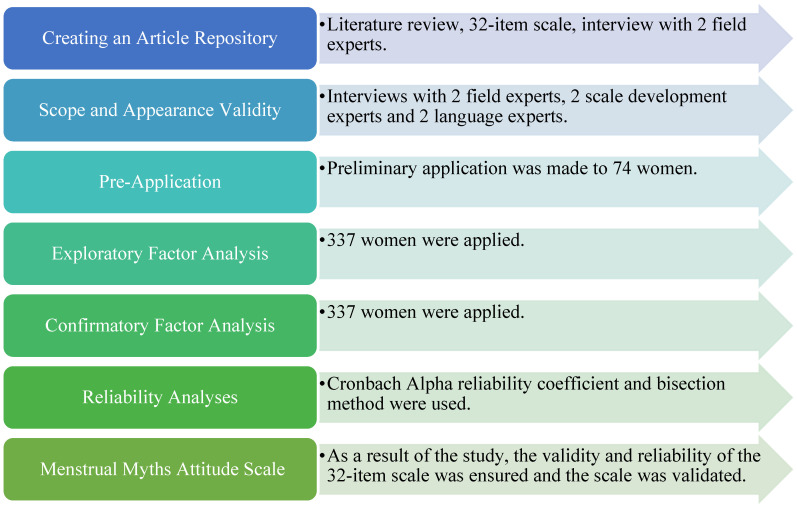
Scale Development Process.

In the first phase of scale development, 32 items were read to 16 women, and they were asked to provide feedback if they did not understand. Because there was no negative feedback during this process, a pre-application was conducted with 74 women to assess comprehension and applicability. Following the pre-application, EFA and CFA analyses were performed to assess the construct validity of the scale. Figure 1 shows the path followed in the scale development process.

### 2.4. Data Analyses

The data were analyzed using IBM SPSS Statistics for Windows, version 22.0 and IBM SPSS AMOS, version 24.0. Descriptive statistics were used to summarize participants’ sociodemographic characteristics and item responses. To examine the construct validity of the scale, exploratory factor analysis (EFA) was performed using principal component analysis with Varimax rotation. The Kaiser–Meyer–Olkin (KMO) measure of sampling adequacy and the Bartlett test of sphericity were used to assess the suitability of the data for factor analysis. Confirmatory factor analysis (CFA) was performed to confirm the factor structure determined by EFA. Model fit was assessed using multiple indices, including the chi-square/degrees of freedom ratio (χ^2^/df), the Root Mean Square Error of Approximation (RMSEA), the Comparative Fit Index (CFI), the Goodness of Fit Index (GFI), the Adjusted Goodness of Fit Index (AGFI), and the Standardized Root Mean Square Residual (SRMR). Internal consistency reliability was assessed using Cronbach’s alpha.

### 2.5. Ethical Considerations

Approval was obtained from Tarsus University Scientific Research and Publication Ethics Committee (Date: 17 December 2021, Number of Decisions: 2021/42). All women in the study were informed about the study on the first page of the data collection tools, and their informed consent was obtained. Necessary explanations were made to the women included in the study in the online form and after the approval was obtained, those who wanted to participate in the study were asked to fill out the online form. In addition, this study was conducted in accordance with the principles of the Declaration of Helsinki and the Personal Data Protection Act.

## 3. Results

In this section, the characteristics of the women participating in the study and the developmental stages of the scale are discussed.

### 3.1. Participant Characteristics

Of the women participating in the study, 57% were married, 59.1% had higher education, 61.1% had income equal to their expenses, and 70.3% lived in the city center (Table 1).

### 3.2. Coefficient Analysis of the Preliminary Application of the Scale

In the scale development process, firstly, the reliability coefficient of the scale items was examined by making a preliminary application. The Cronbach Alpha value obtained as a result of the application is given in Table 2.

When Table 2 is analyzed, it is seen that Cronbach Alpha value is 0.874. A Cronbach’s alpha value between 70 and 90 indicates that the scale is suitable for exploratory factor analysis [22].

### 3.3. Exploratory Factor Analyses

Data obtained during the scale development process was entered into SPPS for exploratory factor analysis. The aim of this analysis was to create a smaller number of significant variables by combining multiple variables. In the study, Direct Oblimin was used for principal components and then rotation. The scree plot table was selected, and the factor loading was set to 0.30 for the analysis. The Kaiser–Meyer–Olkin (KMO) value and the Bartlett’s Test of Sphericity values were examined to determine the suitability of the scale for factor analysis. The Kaiser–Meyer–Olkin (KMO) value is used to determine the adequacy of the sample, and the Bartlett’s Test of Sphericity is used to assess the fit between variables. The KMO value should be between 0.50 and 0.90 [23], and the Bartlett’s Test of Sphericity value should be less than 0.05 [21]. In this study, the KMO value was found to be 0.870 and the Bartlett’s Test of Sphericity value was χ^2^ = 2876.480; df = 496, *p* < 0.05, suitable for factor analysis (Table 3).

According to Table 3, the scale is suitable for factor analysis.

The reference values suggested by Cokluk, Sekercioglu, and Buyukozturk [24] were used in the factor analysis process of the scale. Accordingly, the loading of an item should differ by at least 0.1 from the interfactor loadings, the eigenvalues of the factors should be at least 1, and the common variance ratio of each item should be 0.32. Analysis was conducted according to these criteria, and it was determined that there were no overlapping items.

The principal components method is a frequently used method in exploratory factor analysis [24] and was also used in this study. Varimax (orthogonal) rotation was used in this study, which included multiple dimensions. Pallant [25] reported that item loadings less than 0.30 in the common variance table are not consistent with the items under the common factor. The common loadings and variance explained by the factors are shown in Table 4.

When Table 4 is analyzed, it is seen that a five-dimensional scale with an eigenvalue greater than 1 and explaining 52.074% of the variance is formed. Henson and Roberts [26] state that the explained variance ratio should be 52% and above. In this direction, it is seen that the variance ratio explained by the study is sufficient.

**Figure 2:**
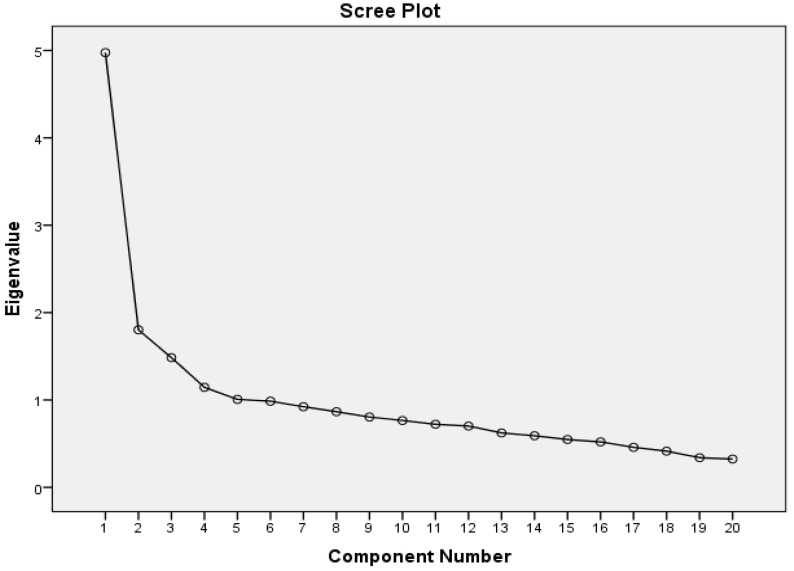
Slope Gradient Graph (Scree Plot).

A scree plot showing the sub-dimensions of the scale is shown in Figure 2.

When the scree slope graph in Figure 2 is examined, it appears that the scale has five dimensions.

According to the data in Table 4 and Figure 2, it is seen that the first factor explains 24.87%, the second factor explains 9.01%, the third factor explains 7.42%, the fourth factor explains 5.72% and the fifth factor explains 5.03% of the total variance. In this respect, it was concluded that the variance explained by the scale was sufficient. Factor names and items in the factors are given in Table 5.

The lowest score from the overall scale was 20, and the highest score was 100. Factors 1, 2, 3, 4, and 5 were derived from the results.

### 3.4. Confirmatory Factor Analysis

**Figure 3:**
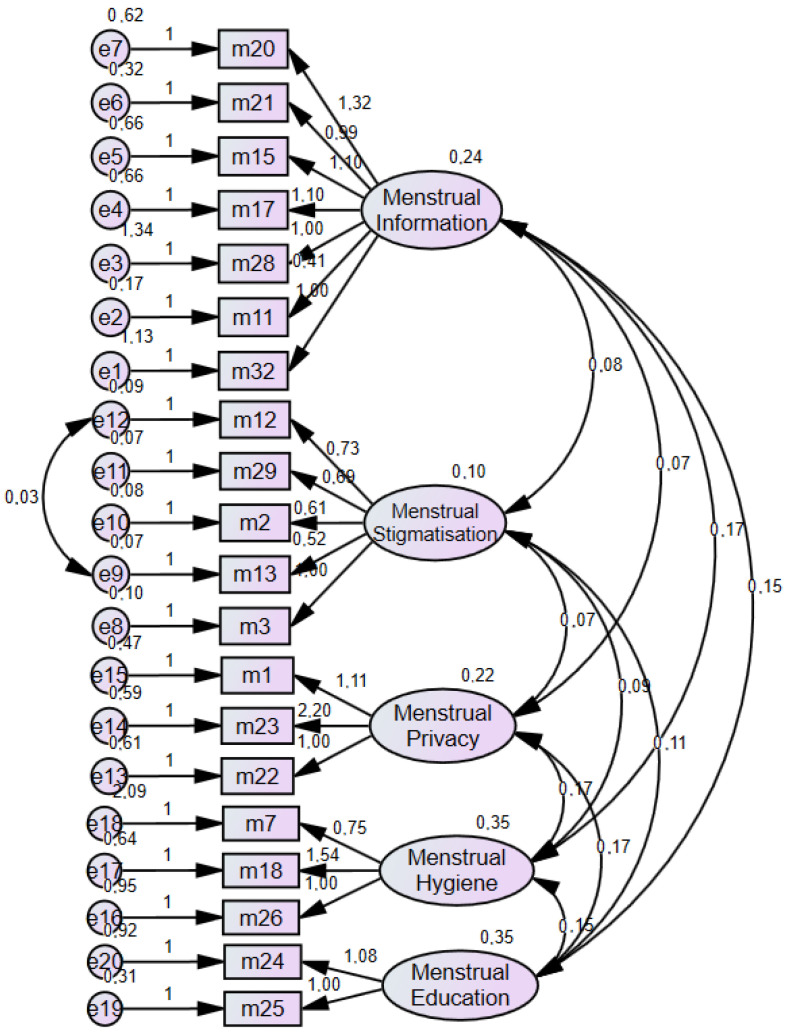
Confirmatory Factor Analysis Path Diagram.

Confirmatory factor analysis (CFA) was conducted to determine whether the scale structure was confirmed, and the path diagram of the CFA is given in Figure 3. In the study, attention was paid to the values being within the range of −2 and +2 for normal distribution. Maximum Likelihood Confirmatory Factor Analysis was performed. In the output section: Standardized estimates, Quadratic multiple correlations, Modification indices, and factor score weights have been selected.

Upon constructing the confirmatory factor analysis (CFA) path diagram depicted in Figure 3, we evaluated the standardized factor loadings, which ranged from 0.293 to 0.802. Note that a minimum threshold of 0.30 is typically accepted [27]. Additionally, covariance was modeled between Items 12 and 13 within the Menstrual Stigmatization subscale.

To assess the model’s adequacy, a variety of goodness-of-fit indices were employed [28], as there is no universal consensus on a single preferred metric [29]. During the CFA, commonly reported indices included χ^2^/df, RMSEA, AGFI, IFI, TLI, GFI, and CFI. For this five-factor, 32-item instrument (see Figure 3), the chi-square likelihood ratio yielded χ^2^/df = 1.655, indicating statistical significance. Other fit statistics were as follows: RMSEA = 0.044, AGFI = 0.910, IFI = 0.928, TLI = 0.913, GFI = 0.932, and CFI = 0.927. These values suggest that the model’s fit falls between “good” and “acceptable” thresholds. Table 6 below compares these CFA outcomes against recommended criteria from the literature [30,31].

According to Table 6, the structure formed as a result of the exploratory factor analysis was confirmed because the scale shows a good and acceptable fit.

### 3.5. Reliability Analyses

Cronbach’s alpha internal consistency coefficient and two-half reliability coefficient calculations were used in the reliability analyses of the items on the scale and are shown in Table 7.

According to the results in Table 7, the scale’s Cronbach’s alpha internal consistency coefficient was 0.794; the first half reliability coefficient was 0.704 and the second half reliability coefficient was 0.705. According to these values, that the scale would appear to have adequate internal and split-half reliability.

Reliability: Internal consistency analyses of the scale were conducted in accordance with international measurement and evaluation standards and COSMIN guidelines [32]. One of the five subscales demonstrated acceptable internal consistency, while Cronbach’s α coefficients were found to be below 0.70 for the other four dimensions. The literature emphasizes that Cronbach’s α is sensitive not only to item homogeneity but also to the number of items and item-to-item correlations [33]. Therefore, a relatively low α is expected, especially in subscales with fewer items. Furthermore, it is known that method effects that may arise from reversed items can negatively affect internal consistency [34]. In this context, low α values were considered not only a weakness in the scale’s reliability but also an indicator of technical limitations arising from the scale’s structural properties and item organization.

Tavsancıl [35] reports reliability coefficients between 0.00 and 0.39 as unreliable (low reliability), between 0.40 and 0.59 as low reliability, between 0.60 and 0.79 as moderate reliability, and between 0.80 and 1.00 as high reliability. The low internal consistency found in the four dimensions of the scale used in this study is a significant limitation in the interpretation of the findings. Low internal consistency values indicate that the homogeneity of the items in these dimensions may be limited, that the small number of items may have reduced the α coefficient, and that methodological problems stemming from reversed items may have had an impact.

## 4. Discussion

This study aimed to develop a reliable and valid scale to determine women’s attitudes toward menstrual myths.

Two criteria are needed to develop a new scale. These criteria are validity and reliability. Construct validity refers to the degree to which a scale accurately represents the construct being measured [36]. In this study, confirmatory and exploratory factor analysis were used to assess construct validity. Factor analysis is widely used to assess construct validity and to test whether items load on different factors [37]. In factor analysis of a scale, the KMO test should first be applied to determine the adequacy and appropriateness of the sample size, and the Bartlett sphericity test should be applied to determine the relationship between variables [36,37,38,39]. In factor analysis, the KMO value must be greater than 0.5 [39,40], and this developed scale found it to be greater than 0.90. Accordingly, the research sample is perfectly adequate for factor analysis. The Bartlett test of sphericity (χ^2^ = 2876.480; SD = 496, *p* < 0.05) indicates that the variables are related and suitable for factor analysis.

Pallant’s common variance table and Catell’s slope plot were used to determine the number of factors for this developed scale. When determining the number of factors on the slope plot, the point at which the graph’s regularity suddenly breaks down should be taken into account. Furthermore, factors with an eigenvalue of at least one should be considered in factor analysis [41]. According to the analysis, five subscales with eigenvalues greater than 1 were identified. These subscales explain 52.074% of the total scale variance. The five-subscale structure is also supported by the scree plot results of our scale. As stated by Buyukozturk [36], if an item has a factor loading below 0.30, it should not be included in the scale. In this study, the factor loadings of the scale, consisting of 20 items and 5 subscales, ranged from 0.41 to 0.80.

When developing a scale, EFA is used to verify the validity of the results obtained in CFA. In CFA, the structure of the scale is examined using X2/SD, RMSEA, AGFI, NFI, TLI, and CFI goodness-of-fit tests. An X2/SD value of five or less from the goodness-of-fit tests indicates a good fit for the tested model [41,42,43]. The X2/SD value was found to be 1.655, indicating a good fit for the scale. Other goodness-of-fit indices for the scale were CFI = 0.927, AGFI = 0.910, and TLI = 0.913. These indices are within the acceptable range. The RMSEA value of the scale is 0.044, indicating an acceptable level of fit. The fit indices are derived from each other, evaluated together, and are closely related to each other [43]. The five-factor structure of the scale was evaluated for fit after EFA, and this was confirmed.

In the reliability analysis of the scale, its internal consistency was examined. The most common method for examining the scale’s internal consistency is the Cronbach’s alpha coefficient, which assesses the agreement between the items [37]. For a scale to demonstrate high reliability, its Cronbach’s alpha value must be between 0.70 and 1.00 [38]. This developed scale has a Cronbach’s alpha value of 0.79, indicating high reliability.

In this study, the five-factor solution was obtained within the set of analytic decisions made for factor extraction. Although multiple decision rules were considered (e.g., scree plot, theoretical coherence), choices regarding extraction/rotation and estimation/correlation type (e.g., ML–Pearson vs. WLSMV–polychoric) may affect the stability of the structure. Moreover, any data-driven model modifications (e.g., correlated residuals) can improve fit while increasing sample-specificity, thereby limiting generalizability. The single-region/convenience sampling frame further warrants caution when extrapolating findings across ages, genders, and contexts. Future work should include cross-validation on an independent sample, test–retest reliability over time, measurement invariance testing (configural/metric/scalar) across key subgroups, and consideration of alternative modeling (e.g., ESEM/bifactor) to more rigorously establish both the stability and generalizability of the five-factor structure.

Menstruation is a uniquely female phenomenon, and myths surrounding it exclude women from many sociocultural spheres. In many societies, even discussing menstruation is a taboo, hindering the development of social and cultural knowledge about the subject. During this period, women are considered unclean, face restrictions in their daily lives, are forbidden from praying, are seen as a source of shame, and are discouraged from physical activity [7]. In many countries, girls are withdrawn from school because they perceive menstruation as the first step toward adulthood and the perception that women will marry. Furthermore, if there are insufficient pads and clean diapers, they cannot attend school that day [44]. Lack of adequate hygiene supplies and the myth that bathing is not allowed make them vulnerable to infection. Furthermore, girls who remain unclean and whose menstrual blood is visible are stigmatized and isolated from school and life [45]. The stress, recurring infections, and stigma they experience harm both their mental and reproductive health [46]. Cultural taboos and myths contribute to the stigmatization of menstruation, restrictions on menstrual hygiene, and hinder women’s awareness and well-being [46]. Menstrual myths negatively impact women of reproductive age physically, socially, psychologically, and spiritually, including the inability to attend school and work [7]. It is essential to understand women’s attitudes toward menstrual myths to prevent reproductive health problems, integrate them into school and work, and prevent social isolation and stigma. This reliable scale facilitates healthcare professionals, educators, researchers, and policymakers to assess the attitudes of women of reproductive age toward menstrual myths. Further evidence can be obtained by repeating the study with different samples and can be used as a decisive tool in initiatives to prevent menstrual myths.

Our findings will contribute to both clinical practice and education as they provide a valid and reliable tool for measuring menstrual myths. Theoretically, the scale provides a framework for systematically examining the cultural and psychosocial dimensions of menstrual myths, while enabling comparative studies across different age groups and cultural contexts in future research.

## 5. Limitations

This study has some limitations. The study was conducted among women of reproductive age in Turkey, which limits its generalizability to women across cultures. The results of the study can only be generalized to the sample that participated in the study. The study does not provide information about the attitudes of men and non-binary individuals toward menstrual myths. Another limitation of the study is that it only included women of reproductive age who use social media. The fact that exploratory factor analysis and confirmatory factor analysis were performed on the same data set is a limitation.

The low internal consistency found in four dimensions of the scale used in this study is a significant limitation in the interpretation of the findings. Low internal consistency values indicate that the homogeneity of the items in these dimensions may be limited, that the small number of items may have reduced the α coefficient, and that methodological problems stemming from reversed items may have had an impact [35]. However, the measurement and evaluation literature indicates that α values in the range of 0.60–0.70 can be tolerated for survey and exploratory studies, depending on the context [47]. Therefore, the low internal consistency values obtained were carefully considered in the interpretation of the results but were not considered to completely eliminate the usability of the scale. Future studies are recommended to increase the number of items in relevant dimensions, revise the item formulations, and, if necessary, revisit the cultural adaptation process.

## 6. Conclusions

In this study, validity and reliability analyses conducted during the scale development process revealed that the scale is a valid and reliable scale for assessing attitudes towards menstrual myths.

Reliability and validity studies can be conducted to ensure that this scale, which consists of twenty items and five subscales, can be used in different countries. With this scale, menstrual myths, false beliefs, and harmful practices existing in society can be scanned. It can be used to produce new research and to determine the attitude of one group before training on menstruation. New scales can be developed to determine menstrual myths and attitudes in different societies.

## Figures and Tables

**Table 1:** Descriptive Statistics of the Sample Group.

		Pre-Application		EFA-CFA	
		n	%	n	%
Marital Status	Married	43	58.1	192	57
	Single	31	41.9	145	43
Education Status	Primary Education	0	0	2	0.6
	Secondary Education	9	12.2	41	12.2
	Higher Education	46	62.2	199	59.1
	Postgraduate	19	25.7	95	28.2
Income Status	My income is less than my expenses	18	24.3	82	24.3
	My income is equal to my expenses	46	62.2	206	61.1
	My income is more than my expenses	10	13.5	49	14.5
Residence	Province	51	68.9	237	70.3
	District	17	23	82	24.3
	Village	6	8.1	18	5.3
Total		74	100	337	100

**Table 2:** Cronbach Alpha Value of the Preliminary Application of the Scale.

Cronbach Alpha	Mean	Variance	Item Number
0.874	1.864	0.415	32

**Table 3:** Kaiser–Meyer–Olkin and Bartlett’s Sphericity Tests.

Kaiser–Meyer–Olkin Measurement of Sampling Adequacy.		0.870
Bartlett’s Sphericity Test	Chi-Square	2876.480
	sd	496
	*p*	0.000

**Table 4:** Common Variances, Item Loadings, and Explained Variance Ratios of Scale Items.

			Common Variance	Factor Load Values
1st dimension	m20	Drinking cold drinks during menstruation negatively affects menstruation.	0.567	0.731
	m21	It is harmful to eat certain foods (pickles, dairy products such as curd, etc.) during menstruation.	0.556	0.707
	m15	Physical activity/exercise during menstruation increases dysmenorrhoea.	0.411	0.587
	m17	During menstruation, the woman’s odor changes and is noticeable from the outside.	0.414	0.540
	m28	A man who has sexual intercourse with a woman during menstruation becomes infected.	0.353	0.537
	m11	After menarche (first menstruation), every girl is ready for marriage.	0.314	0.504
	m32	If a woman experiences discomfort during menstruation, the doctor should be preferred.	0.252	0.411
2nd dimension	m12	Menstruation is a shameful condition.	0.608	0.727
	m29	During menstruation, the woman damages everything she touches.	0.565	0.684
	m2	During menstruation, the woman should stay in a separate room (home/shelter/environment).	0.472	0.673
	m13	Menstruation is a punishment given to the woman.	0.528	0.663
	m3	When a woman menstruates she becomes unclean.	0.619	0.659
3rd dimension	m1	A woman should hide her menstruation from everyone and live in secret.	0.649	0.778
	m23	Menstruation is not a subject to be discussed in the presence of men.	0.692	0.727
	m22	When talking about menstruation in public, women should use symbols (special expressions) (sick, period, monthly flower, motherland is crying blood, cherry day, etc.).	0.529	0.653
4th dimension	m7	After menstruation, women should take a bath or a purification ritual (oil, water, salt, fire) to cleanse themselves.	0.577	0.732
	m18	Some personal care practices (hair coloring/cutting, nail cutting, hair removal, etc.) should not be performed during menstruation.	0.571	0.601
	m26	During menstruation, women should not pray, enter places of worship or visit holy places.	0.465	0.598
5th dimension	m24	Women themselves should obtain information about menstruation.	0.714	0.807
	m25	Menstruation education should only be given to women.	0.560	0.610
	Total Explained Variance			%52.074

**Table 5:** Factor Names and Items in the Factors of the Attitude Towards Menstrual Myths Scale.

Factor	Factor Name	Related Items
First factor	Menstrual Information	m20, m21, m15, m17, m28, m11, m32
Second factor	Menstrual Stigmatization	m12, m29, m2, m13, m3
Third factor	Menstrual Privacy	m1, m23, m22
Fourth factor	Menstrual Hygiene	m7, m18, m26
Fifth factor	Menstruation Education	m24, m25

**Table 6:** Confirmatory Factor Analysis Fit Indices.

Indexes	Reference Value		Measurement	Result
	Acceptable Fit	Good Fit		
CMIN/DF	3 < χ^2^/sd ≤ 5	0 < χ^2^/sd ≤ 3	1.655	Good Fit
RMSEA	0.05 ≤ RMSEA ≤ 0.08	0 ≤ RMSEA ≤ 0.05	0.044	Good Fit
AGFI	0.85 < AGFI ≤ 0.89	0.90 < AGFI ≤ 1	0.910	Good Fit
IFI	0.90 < IFI ≤ 0.94	0.95 < IFI ≤ 1	0.928	Acceptable Fit
TLI	0.90 < TLI ≤ 0.94	0.95 < TLI ≤ 1	0.913	Acceptable Fit
GFI	0.85 < GFI ≤ 0.89	0.90 < GFI ≤ 1	0.932	Good Fit
CFI	0.90 < CFI ≤ 0.94	0.95 < CFI ≤ 1	0.927	Acceptable Fit

**Table 7:** Reliability Analysis for Internal Consistency, Two Halves and Subscales.

Test Type		Cronbach Alpha
Internal Consistency Coefficient		0.794
Menstrual Information		0.68
Menstrual Stigmatization		0.76
Menstrual Privacy		0.66
Menstrual Hygiene		0.48
Menstruation Education		0.55
Split-Half Coefficient	Part 1	0.704
	Part 2	0.705

## Data Availability

The datasets used and/or analyzed during the current study are available from the corresponding author on reasonable request.

## Abbreviations

The following abbreviations are used in this manuscript:

EFA	Exploratory Factor Analysis
CFA	Confirmatory Factor Analysis
KMO	Kaiser–Meyer–Olkin
